# Molecular and HPLC-based approaches for detection of aflatoxin B_1_ and ochratoxin A released from toxigenic *Aspergillus* species in processed meat

**DOI:** 10.1186/s12866-021-02144-y

**Published:** 2021-03-14

**Authors:** Abdelazeem M. Algammal, Mahmoud E. Elsayed, Hany R. Hashem, Hazem Ramadan, Norhan S. Sheraba, Eman M. El-Diasty, Sarah M. Abbas, Helal F. Hetta

**Affiliations:** 1 Department of Bacteriology, Immunology and Mycology, Faculty of Veterinary Medicine, Suez Canal University, Ismailia, 41522 Egypt; 2 Department of Microbiology and Immunology, Faculty of Pharmacy, Fayoum University, Fayoum, 63514 Egypt; 3 Hygiene and Zoonoses Department, Faculty of Veterinary Medicine, Mansoura University, Mansoura, 35516 Egypt; 4 VACSERA, The Holding Company for Biological Products and Vaccines, Giza, 12511 Egypt; 5 Animal Health Research Institute, Dokki, Giza, 12618 Egypt; 6 Department of Medical Microbiology and Immunology, Faculty of Medicine, Assuit University, Assuit, 71515 Egypt

**Keywords:** *A. flavus*, *A. ochraceus*, Meat-products, HPLC, Aflatoxin B_1_, Ochratoxin A, Gene sequencing

## Abstract

**Background:**

Meat-products are considered an enriched media for mycotoxins. This study aimed to investigate the prevalence of toxigenic *Aspergillus* species in processed meat samples, HPLC-quantitative measurement of aflatoxin B_1_ and ochratoxin A residues, and molecular sequencing of *aflR1* and *pks* genes. One hundred and twenty processed beef meat specimens (basterma, sausage, and minced meat; *n* = 40 for each) were collected from Ismailia Province, Egypt. Samples were prepared for total mold count, isolation, and identification of *Aspergillus* species. All samples were analyzed for the production of both Aflatoxin B_1_ and Ochratoxin A mycotoxins by HPLC. Molecular identification of *Aspergillus flavus* and *Aspergillus ochraceus* was performed using PCR amplification of the internal transcribed spacer (ITS) region; furthermore, the *aflR1* and *pks* genes were sequenced.

**Results:**

The total mold count obtained from sausage samples was the highest one, followed by minced meat samples. The prevalence of *A. flavus* was (15%), (7.5%), and (10%), while the prevalence of *A. ochraceus* was (2.5%), (10%), and (0%) in the examined basterma, sausage, and minced meat samples, respectively. Using PCR, the ITS region was successfully amplified in all the tested *A. flavus* and *A. ochraceus* strains. Aflatoxin B_1_ was detected in six basterma samples (15%). Moreover, the ochratoxin A was detected only in four sausage samples (10%). The *aflR1* and *pks* genes were amplified and sequenced successfully and deposited in the GenBank with accession numbers MF694264 and MF694264, respectively.

**Conclusions:**

To the best of our knowledge, this is the first report concerning the HPLC-Molecular-based approaches for the detection of aflatoxin B_1_ and ochratoxin A in processed beef meat in Egypt. The production of aflatoxin B_1_ and ochratoxin A in processed meat constitutes a public health threat. Aflatoxin B_1_ is commonly associated with basterma samples. Moreover, ochratoxin A was detected frequently in sausage samples. The routine inspection of mycotoxins in processed meat products is essential to protect human consumers.

## Background

Beef meat and meat products are considered the most desirable and favorable food in Egypt. Beef meat is characterized by a high nutritional value due to its high content of essential amino acids, minerals, fats, and vitamins. In 2019, Egypt imported beef meat with the following values: 982 million US$ from Brazil (65% market share), 466 million US$ from India (31% market share), 16.2 million US$ from Colombia, 8.25 million US$ from Australia, 5.08 million US$ from New Zealand, and 2.4 million US$ from the USA [1, 2]. Basterma and sausage are processed meat products prepared from meat with the addition of food additives. The contamination of beef meat and meat products with mycotoxins constitutes public health hazards to the consumers. Depending on the type and predilection sites of the mycotoxins, different symptoms in humans have been determined including hepatotoxicity, liver cancer, nephrotoxicity, immunosuppression, mutagenicity, nervous and hormonal system disturbance [3].

Mycotoxins are commonly produced by the following species: *Aspergillus*, *Fusarium,* and *Penicillium* [4]. Many studies on mammals and poultry reported that: mycotoxins have mutagenic, hepatotoxic, carcinogenic, teratogenic, immunosuppressive, nephrotoxic, and embryotoxic effects [5]. Although single mold species could release more than one type of mycotoxin, one mycotoxin could be produced by different mold species. Globally, aflatoxins (AFs) are the most medically significant mycotoxins that contaminate human and animal foodstuff. According to the International Agency for Research on Cancer (IARC), aflatoxins, produced primarily by *A. flavus* have been confirmed as carcinogenic agents that mainly affects liver causing hepatocellular carcinoma [6, 7]. The treatment of aflatoxins with ultra-high temperature (UHT), roasting, pasteurization, baking, and cold storage cannot destruct them because aflatoxins are heat-stable. Numerous types of AFs exist naturally; but, the most potent types are; B_1_, B_2_, G_1_, and G_2_ [8]. Ochratoxin A (OTA) is a secondary metabolite produced mainly by *Aspergillus* species especially, *A. ochraceus* when the environmental and storage conditions are optimum for the growth and multiplication of these fungi, such as in tropical and subtropical regions [9, 10]. The major types of OTA are A, B, and C, that pose a significant threat to human and animal health [11].

Contamination of meat and meat products with molds commonly results from contaminated equipment or air. The mold contamination results in unfavorable alterations in the meat and meat products that causes severe infections, mycotoxicosis, and allergic reactions to consumers [12]. Humans are subjected to OTA either by ingestion, inhalation, or skin contact. Certain types of foods are considered the main source for OTA such as; coffee beans, grapes, wine, beef meat, beef meat products, poultry, pork, fish, cheese, and eggs [13].

Processed meat products are considered excellent substrate for mycotoxigenic fungal proliferation/colonization and the subsequent mycotoxin production; due to the mold contamination that occurs during the handling, manufacturing, and storage [14]. Various species of fungi produce hundreds of toxic metabolites. Globally, meat products are considered a common source of mycotoxins [15–19]. The mold contamination of meat and meat products results in severe illness in humans and animals due to the production of mycotoxins such as aflatoxins and ochratoxin A (OTA) [7]. Several studies from different countries have reported the occurrence of mycotoxins in both fresh and processed meat. Despite this topic has gained much attention in the last years, the European Regulation does not set specific limits for aflatoxins and ochratoxins in meat and processed meat products. The existence of mycotoxins in meat and meat products is considered a public health threat that magnifiy the need for more investigations concerning the detection of mycotoxins in such type of food [20–26].

This study aimed to investigate the prevalence of toxigenic *Aspergillus* species in processed meat samples, HPLC-quantitative measurement of aflatoxin B_1_ and ochratoxin A residues, and molecular sequencing of aflatoxin regulatory gene (*aflR1*) and polyketide synthase gene (*pks*). To the best of our knowledge, this is first study combined HPLC and Molecular assays for the detection of aflatoxin B_1_ and ochratoxin A in processed beef meat in Egypt.

## Materials and methods

### Samples collection and processing

A total of 120 processed beef meat specimens (basterma, sausage, and minced meat; *n* = 40 for each type) were collected randomly from licensed retail markets that gained good hygiene practice (GHP) in 4 different localities in Ismailia Province, Egypt. The collected specimens were labeled, placed into polyethylene sterile bags, and rapidly transported under complete aseptic conditions in an icebox to the Microbiology laboratory, Animal Health Research Institute, Egypt. For each specimen, 25 g were minced aseptically in a grinder through a 4 mm sterilized plate diameter (AC110V, China). In Egypt, the basterma is prepared from beef meat (3–5 cm thickness), salt, and other additives (ground fenugreek seed, ground paprika, cumin, black pepper, cayenne pepper, and garlic). Moreover, beef sausage is mostly produced from beef meat, fat tissues, salt, spices mixture (fennel, black pepper, cubeb, Nutmeg, cinnamon, cumin, and clovers), garlic, starch, and sodium glutamate. Besides, minced meat is prepared by grinding beef meat and fat (75%:25%).

### Mold enumeration, isolation, and identification of *Aspergiluus* spp.

For propagation of mold associated with food spoilage, each minced sample was mixed with 225 mL of sterile peptone water (0.1%), then ten-fold serial dilutions were performed as previously reported by Downes and Ito [27]. Briefly, 1 mL of the processed dilution was poured into duplicated sterile Petri dishes, and then gently mixed with Dichloran Rose Bengal Chloramphenicol agar (Oxoid, UK). The inoculated plates were incubated up to 1 week at 25 °C and then examined for the mold growth and enumeration expressed as CFU/g. The suspected colonies were inoculated onto Sabouraud Dextrose slant agar (Oxoid, UK), and incubated at room temperature for up to 5 days for further mycological examination. The identification of suspected colonies was carried out depending upon the macroscopical examination; growth rate, texture, diameter, color, and characters of examined colonies, and the microscopical examination using lactophenol blue staining to investigate the morphological characters including; the conidial stage and head, sclerotia production, conidia, and conidiophore as previously described by Pitt and Hocking [28].

### Extraction and HPLC-quantitative measurement of aflatoxin B_1_ and ochratoxin A residues

The chemicals and reagents used for extraction and HPLC-quantitative measurement of mycotoxins in the processed meat samples were purchased from Sigma (Sigma, Germany). Phosphate-buffered saline (PBS) was prepared by dissolving 8 g of NaCl, 0.2 g of KCl, 0.2 g of KH_2_PO_4_, and 1.2 g of Na_2_HPO_4_ in 1000 mL of water. The pH for PBS was adjusted to 7.0 with 0.1 M HCl.

Ten grams of each examined specimen were homogenized with 40 mL of acetonitrile: water (60:40, v/v) and 0.2 g NaCl for 90 s, then blended by a magnetic stirrer for 10 min. The filtration of the mixture was carried out through fast filtering Whatman No. 1 filter paper (Whatman Inc., Clifton, NJ, USA). Four mL of the filtrate were diluted with 44 mL of 2% tween-20-PBS solution in a 50 mL Erlenmeyer flask. Then, the filtrate was cleaned up using liquid/liquid extraction method as follows: 0.5 mL aliquot of filtrate was mixed with 0.5 mL acetonitrile, then 0.5 mL of the mixture was pipetted into an Alltech 1.5 mL Extract-Clean reservoir packed with 200 mg basic aluminum oxide (9 mm high-layer adsorbent). The quantitative detection of the mycotoxins was performed by HPLC system (Thermo Fisher Scientific, Waltham, MA 02451, USA) using 100 μL of the extract as previously described by Herzallah [29].

The fluorescence-detector was adjusted to an excitation and an emission wavelength of 365 nm and 435 nm, respectively. Concerning the validation and quality assurance of the quantitative measurement of aflatoxins; a seven-point standardization curve was conducted using the following concentrations: (0.1, 0.5, 1, 2, 5, 10, and 20 μg/kg) for aflatoxin B_1_. Moreover, the signal-to-noise approach was used to detect the limits of quantification (LOQ) and the limits of detection (LOD).

To ensure the accuracy of the test, approximately 25 g aflatoxins-free sample (for each sample type) was spiked with aflatoxin B_1_ at levels of 3, 5, and 10 *μ*g/ kg. The spiked samples were examined using the HPLC, followed by the estimation of both the recovery and standard deviation. The protocol was performed in three replicates. For the validation and quality assurance of the quantitative measurement of ochratoxins, a five-point standardization curve was conducted using the following concentrations: 0.5, 2, 5, 10, and 30 μg/kg.

Besides, the signal-to-noise approach was used to detect the limits of quantification (LOQ) and the limits of detection (LOD). To ensure the accuracy of the test, about 25 g ochratoxin A-free sample (for each sample type) was spiked with ochratoxin A at the levels of 1, 5, and 20 *μ*g/kg. The assay was performed in three replicates. The recovery rate for aflatoxin B_1_ and ochratoxin A was 90 and 92%, respectively [29, 30].

### Molecular identification of *A. ochraceus* and *A. flavus* by amplification of internal transcribed spacer (ITS) region

Colonies with distinct phenotypic characters were used for DNA extraction. The DNA extraction was performed using the Patho Gene-SpinTM DNA/RNA Extraction kit (iNtRON cat. No. 17154, Korea). ITS region was amplified by PCR using the forward primer ITS1 5′-TCCGTAGGTGAACCTGCGG-3′ and the reverse primer ITS4 5′-TCCTCCGCTTTATTGATATG − 3′ (Sigma, Germany) with variable expected amplicon size (700–800 bp) [31]. PCR mixtures consisted of 50 μL containing; 25 μL master mix (Bioline, cat. BIO-25049, England), one μL each primer, and 30 to 80 ng of genomic DNA or distilled water (as a negative control). PCR was programmed as follow: 94 °C for 4 min followed by 35 cycles of 94 °C for 1 min, 56 °C for 1 min and 72 °C for 1 min. A final extension step was done at 72 °C for 10 min. PCR amplicons were visualized by gel electrophoresis (1.5%).

### Amplification and sequence analysis of aflatoxin regulatory gene (*aflR1*) of *A. flavus*

PCR amplification for the *aflR1* gene was performed in six toxigenic isolates of *A.flavus* using the forward primer *AflR*-1F 5′-AAGCTCCGGGATAGCTGTA-3′ and the reverse primer *AflR*-2R 5′-AGGCCACTAAACCCGAGTA − 3′ for the identified isolates with an expected amplicon size of 1079 bp [32]. Fifty μL volume of PCR was done as follow: initial denaturation at 95 °C for 10 min followed by 30 cycles of 94 °C for 30 s, 55 °C for 45 s, and 72 °C for 75 s. A final extension step was carried out at 72 °C for 10 min. As the retrieved *A. flavus* isolates revealed harmony in their phenotypic characteristics, the PCR product of one randomly selected isolate was purified with the Gene JETPCR purification kit (Thermo Scientific, Cat. K0701). PCR amplicons were sequenced in both directions using the Applied-Biosystem Automated 3730XL DNA sequencer (Macrogen, Seoul, South Korea). The obtained sequences were deposited in GenBank with accession No. MF094441, and then analyzed using the BLASTn tool at the National Center of Biotechnology Information. The evolutionary history was inferred by using the Maximum Likelihood method based on the Tamura-Nei model [33]. The evolutionary analyses were conducted using MEGA6 software (http://www.megasoftware.net/) [34].

### Amplification and sequence analysis of polyketide synthase gene (*pks*) of *A. ochraceus*

Two sets of primers were used for PCR amplification of the *pks* gene (Table 1) in 4 toxigenic isolates of *A.ochraceus*. Fifty μL volume PCR was carried out as follows: initial denaturation at 94 °C for 4 min followed by 35 cycles of 94 °C for 40 s, 58 °C for 40 s, and 72 °C for 40 s. A final extension step was carried out at 72 °C for 10 min. Meanwhile, the recovered *A. ochraceus* isolates showed harmony in their phenotypic characteristics: the PCR product of one randomly selected isolate was purified with was purified by the Gene JETPCR purification kit (Thermo Scientific, Cat. K0701). PCR amplicons were sequenced in both directions using the Applied-Biosystem Automated 3730XL DNA sequencer (Macrogen, Seoul, South Korea). The obtained sequences were deposited in GenBank with accession No. MF694264, and then were analyzed using the BLASTn tool at the National Center of Biotechnology Information. The evolutionary history was inferred by using the Maximum Likelihood method based on the General Time Reversible model [36]. The evolutionary analyses were performed using MEGA6 software (http://www.megasoftware.net/) [34].

**Table 1:** Primers used for the amplification of *pks* gene of *A. ochraceus*

Primer	Oligonucleotide sequence	Expected amplicon Size (pb)	Reference
AoLc35-12 L	5′-GCCAGACCATCGACACTGCATGCTC-3’	520	[35]
AoLc35-12R	5′-CGACTGGCGTTCCAGTACCATGAGCC-3’		
AoOTA-L	5′-CATCCTGCCGCAACGCTCTATCTTTC-3’	690	
AoOTA-R	5′-CAATCACCCGAGGTCCAAGAGCCTCG-3’		

### Statistical analysis

The statistical analyses carried out using GraphPad Prism version 8.0.1 (244) (San Diego, CA, USA). All results were elaborated as mean together with standard deviation (SD). The Chi-square was implemented to analyze the data; the significance level was (*P* < 0.05).

## Results

### Total mold counts (CFU/g) in meat products samples

Total mold count (CFU/g) obtained from sausage samples was the highest one (2.9 × 10^2^ ± 0.91 × 10^2^), followed by minced meat samples (1.74 × 10^2^ ± 0.52 × 10^2^), and basterma samples (0.79 × 10^2^ ± 0.31 × 10^2^). Statistically, there is a significant difference in the total mold count among various examined samples (*P* < 0.0001) (Table 2).

**Table 2:** Total mold count in examined meat products specimens (CFU/g)

Meat products	Total mold count (CFU/g)			
	Minimum	Maximum	Mean(±SD)	***P*** value
Basterma	36	140	0.79 × 10^2^ ± 0.31 × 10^2^	*P < 0.0001
Sausage	103	380	2.9 × 10^2^ ± 0.91 × 10^2^	
Minced meat	70	249	1.74 × 10^2^ ± 0.52 × 10^2^	

*Significant, (*P* < 0.05)

### The phenotyic chracterstics and prevalence of *A. flavus* and *A. ochraceus* in the examined meat products samples

**Fig. 1:**
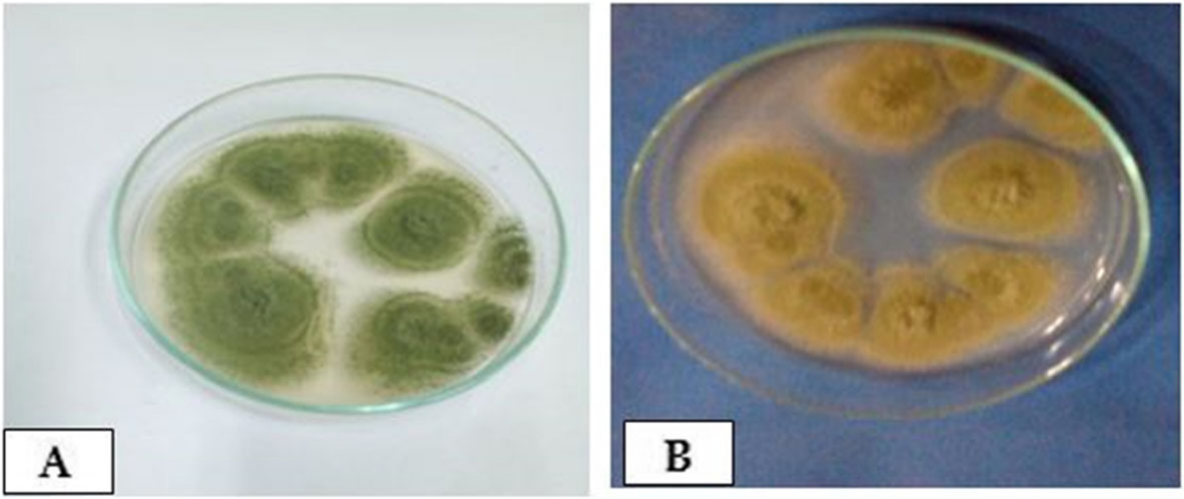
*A. flavus* and *A. ochraceus* colonies on SDA:. **a**. *A. flavus*: white soft velvety colonies that turn yellowish-green, a pigment of the conidial spores. **b**. *A. ochraceus*: white soft velvety colonies that turn yellow-gold conidia, a pigment of the conidial spores

**Fig. 2:**
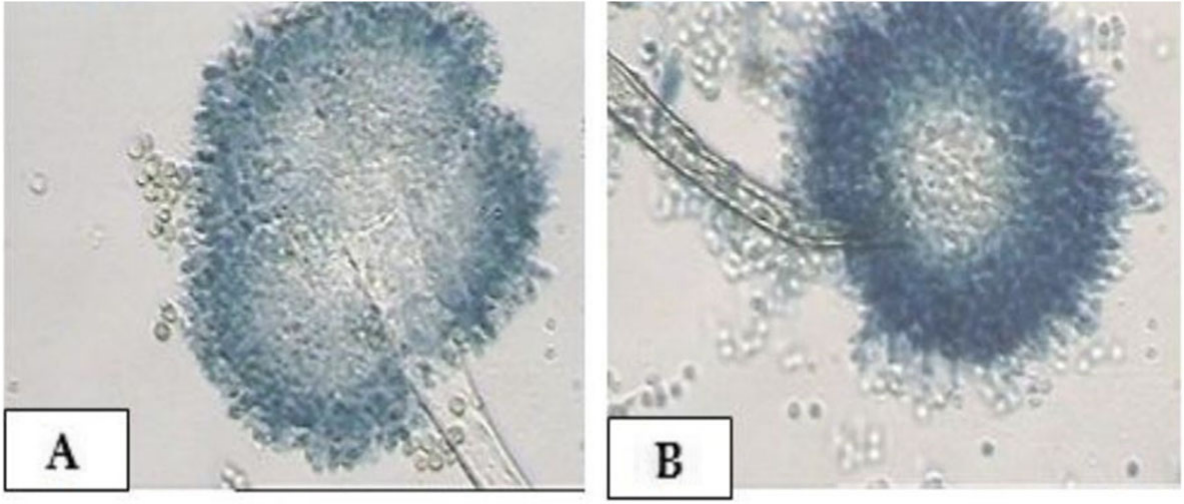
Microscopical examination of *A. flavus* and *A. ochraceus* (40×): **a**. *A. flavus*: conidial heads are radiate to loosely columnar with age. **b**. *A. ochraceus*: distinct globose conidial head

Concerning the phenotypic characteristics of the recovered isolates, the colonies of *A. flavus* are characterized by a white soft velvety surface that becomes raised and turned floccose at the center after few days. The colonies produced yellowish-green and olive conidia during the sporulation. The conidia cover the entire surface of the colonies except for the edges, where a white border was produced. Sclerotia produced in white color then became deep brown. The diameter of *A. flavus* colonies ranged from 50 to 70 mm. Moreover, the colonies of *A. ochraceus* grow rapidly and are characterized with white soft velvety surfaces then turned in a characteristic yellow-gold color and have distinct globose conidial heads. The conidiophores have a powdery form that could be observed by the naked eye. Furthermore, the mycelium is submerged mainly in the agar media, and the conidial heads are commonly arranged in zones. The reverse appearance of the petri dish is mainly brownish. The diameter of *A. ochraceus* colonies ranged from 45 to 55 mm. (Figs. 1 and 2).

In the present study, the prevalence of *A. flavus* was (15%), (7.5%), and (10%) in the examined basterma, sausage, and minced meat samples, respectively. Moreover, the prevalence of *A. ochraceus* was (2.5%), (10%), and (0%) in the examined basterma, sausage, and minced meat samples, respectively (Table 3).

**Table 3:** The prevalence of *A. flavus* and *A. ochraceus* isolates in the examined meat product samples

Isolated ***Aspergillus*** species	Basterma (***n*** = 40)		Sausage (***n*** = 40)		Minced meat (***n*** = 40)	
	Number of isolates	%	Number of isolates	%	Number of isolates	%
*A. flavus*	6	15	3	7.5	4	10
*A. ochraceus*	1	2.5	4	10	0	0

### Prevalence of aflatoxin B_1_ and ochratoxin A in the examined meat products samples

Concerning the occurrence of aflatoxin B_1_ in the examined samples, it could be detected and quantified only in six basterma samples (15%). The minimum detected level was 16.5 μg/kg, while the maximum level of detection was 26.6 μg/kg with a mean value of 21.80 ± 3.823 μg/kg. Moreover, the examined minced meat and sausage samples were negative for aflatoxin B_1_.

Regarding the occurrence of ochratoxin A in the examined samples, it was detected only in four sausage samples (10%). The minimum detected level was 3.8 μg/kg, while the maximum level of detection was 17 μg/kg with a mean value of 10 ± 2.9 μg/kg. Furthermore, the examined basterma and minced meat samples were negative for ochratoxin A. Statistically, the production of aflatoxin B_1_ in basterma samples is significantly different from sausage and minced meat (*P* < 0.0001). Moreover, the production of ochratoxin A in sausage samples is significantly different from basterma and minced-meat (*P* = 0.0041; *P* < 0.05) (Table 4).

**Table 4:** Prevalence of aflatoxin-B_1_ and ochratoxin A in the examined meat product samples (μg/kg)

Meat Products	Aflatoxin B_**1**_					Ochratoxin A				
	No. of + ve samples	%	Range (μg/kg)	Mean ± SD	***P*** value	No of + ve samples	%	Range (μg/kg)	Mean ± SD	***P*** value
Basterma *N* = 40	6	15	16.5–26.6	21.80 ± 3.823	*P < 0.0001	0	0	0	0.0 ± 0.0	*P = 0.0044
Sausage *N* = 40	0	0	0	0.0 ± 0.0		4	10	3.8–17	10 ± 2.9	
Minced meat *N* = 40	0	0	0	0.0 ± 0.0		0	0		0.0 ± 0.0	

*Significant, (*P* < 0.05)

### Molecular identification of *A. flavus* and *A. ochraceus* by PCR amplification of internal transcribed spacer (ITS) region

The genetic identification of the recovered *A. ochraceus* and *A. flavus* isolates was carried out usig PCR amplification of internal transcribed spacer (ITS) region. The PCR revealed that the ITS region was successfully amplified in all tested *A. flavus* and *A. ochraceus* isolates and giving the specific molecular size.

### Amplification and sequence analysis of aflatoxin regulatory gene (*aflR1*) of *A. flavus*

**Fig. 3:**
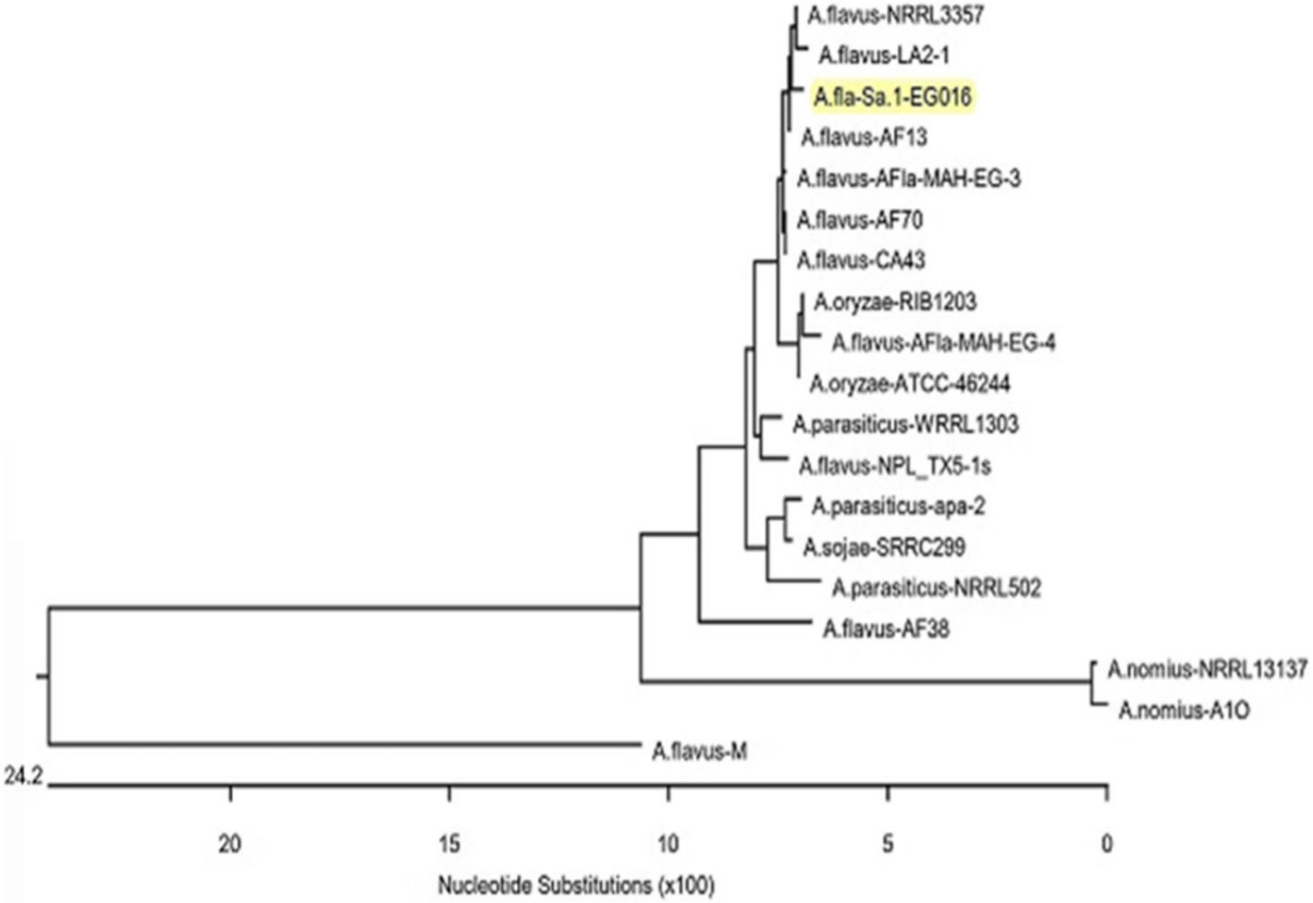
Phylogenetic analysis of the *aflR1* gene using maximum likelihood method. The tree was generated based on the *aflR* gene nucleotide sequence; it illustrates the phylogenetic position of the retrieved strain (A.fla-Sa.1-EG016) with respect to other strains deposited in Genbank where the topology of the joining tree of the *aflR* gene sequence is almost the same. The phylogenetic tree was created by MEGA6 (http://www.megasoftware.net/)

Using PCR, the *aflR1* gene was amplified successfully in six *A. flavus* strains isolated from the examined basterma samples (~ 1079 bp). The PCR products were sent for sequencing, and the retrieved sequences were deposited in the GenBank with accession number: MF094441. According to the blastn tool at the NCBI, the identity was 99.73 with query covered 100% percentage to the other *aflR1* genes deposited in the Genebank database (Fig. 3).

### Amplification and sequence analysis of polyketide synthase gene (*pks*) for the of *A. ochraceus*

**Fig. 4:**
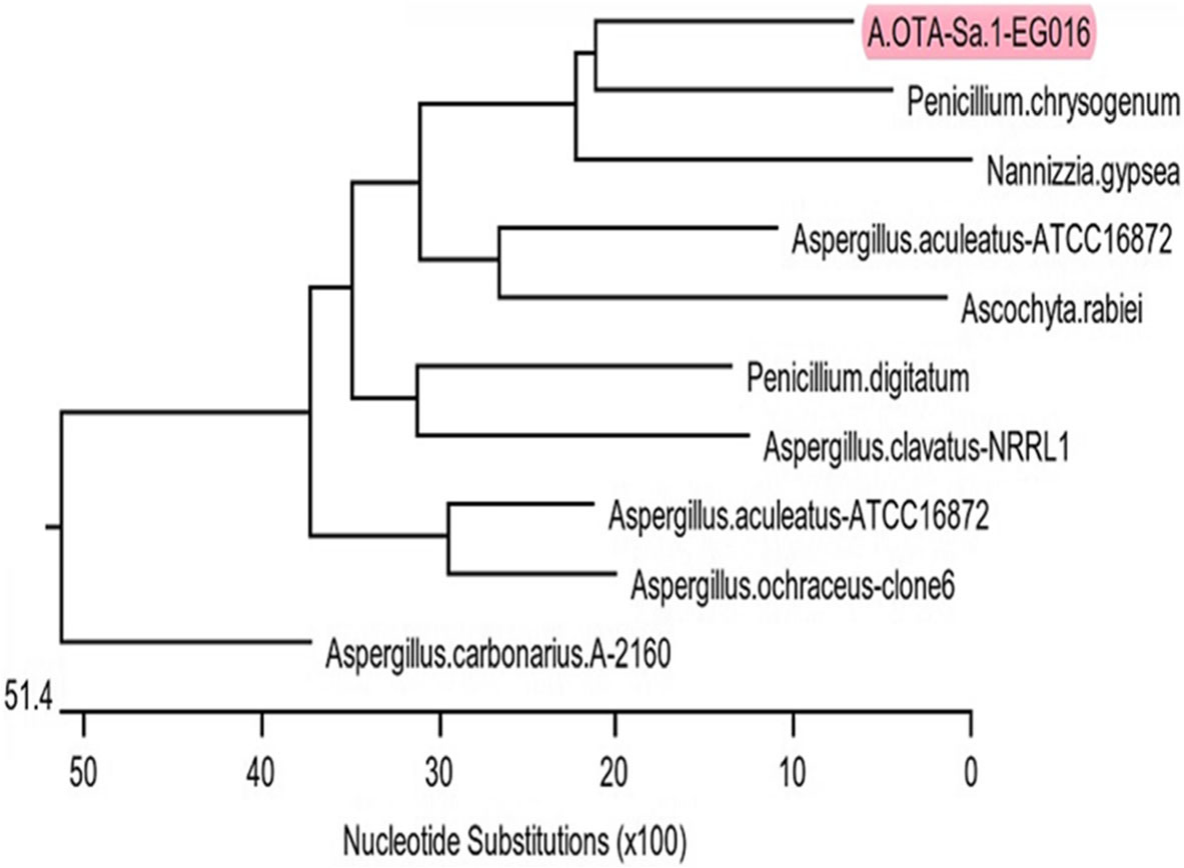
The phylogenetic analysis of *pks* gene using the maximum likelihood method. The trees was generated based on *pks* gene nucleotide sequence; it illustrates the phylogenetic position of the retrieved strain (AOTA-Sa.1-EGO16) with respect to other strains deposited in Genbank where the topology of the joining tree of the *pks* gene sequence is almost the same. The phylogenetic tree was created by MEGA6 (http://www.megasoftware.net/)

The PCR proved that the *pks* gene was amplified successfully in four *A. ochraceus* strains isolated from sausage samples. The PCR products were sent for sequencing, and the recovered sequences were deposited in the GenBank with accession number: MF694264. According to the blastn tool, the similarity was 100 with query covered 100% percentage to other *pks* genes deposited in the Genebank database (Fig. 4).

## Discussion

Our results showed that the highest mean count (CFU/g) was in sausage (2.9 × 10 ^2^ ± 0.91 × 10^2^), followed by 1.74 × 10^2^ ± 0.52 × 10^2^ for minced meat, and 0.79 × 10^2^ ± 0.31 × 10^2^ for basterma. A previous study reported that the count was 2.8 × 10^2^ ± 37.4 for basterma samples [37]. Moreover, Mousa et al. [38], and Ebraheem and Mohamed [39] reported that the mold count was 1.22 × 10^2^ ± 0.49 × 10^2^ and 2.4 × 10^2^ ± 0.27 × 10^2^ in basterma samples, respectively. Besides, a previous investigation [40] reported that the mean total mold count was 2.26 × 10^2^ ± 0.58 × 10^2^ in sausage samples. Molds are commonly detected inside or on the surface of definite aged, preserved meats, especially fermented sausage and could be metabolically active during the prolonged curing time and the ripening time of sausage. Therefore, fungi extremely affect the appearance, flavor, and quality of these meats. Moreover, the presence of cured salts, low water activity condition (a_w_), pH falls due to fermentation processes in sausage samples, and the ability of molds to withstand high concentrations of cured meat make sausage a favorable environment for mold growth [41–43].

In the present study, ten retrieved isolates were toxigenic (10/18, 55.55%) including six *A. flavus* strains (originated from the basterma samples) harbored the aflatoxin B_1_ and four *A. ochraceus* strains (isolated from sausage samples) harbored the ochratoxin A. Besides, *A. parasiticus* and *A. nomius* were not detected during the isolation and identification. In Egypt, *A. flavus* and *A. ochraceus* are the most predominant species [38]. Our findings revealed that *A. flavus* is the most predominant mold species isolated from the processed meat samples. Ebraheem and Mohamed [39] reported that the most predominant isolated mold species from basterma and luncheon samples was *A. flavus.* Besides, Makhlouf et al. [44] reported that *Aspergillus* was the chief genus found in the examined spices samples that used in the meat processing; *A. flavus* and *A. ochraceus* were the most predominant species, which is also in agreement with the findings reported by previous studies in Morocco, India, and Brasil [45–48]. In the current study, the prevalence of *A. flavus* was higher than *A. ochraceus* in the examined processed beef meat samples. *A.flavus* is a ubiquitous microorganism widely distributed in nature, soil, and different types of foods*. A. flavus* is an opportunistic microorganism characterized by a broad host range. Besides, *A. flavus* commonly contaminates most of the food additives used in meat processing [39, 49].

In the present study, the highest percentage of toxigenic isolated *Aspergillus* species was recorded in basterma samples followed by sausage samples. Moreover, no toxigenic *Aspergillus* species was detected in minced meat samples. Zohri et al. reported that two out of the four strains of *A. flavus* could produce aflatoxin B_1_ in the examined sausage samples [50]. Besides, a previous study reported that aflatoxins were detected in 15% of minced meat samples and 10% of fresh sausage [51]. Mycotoxins production is mainly affected by the type and composition of meat products, feed additives, the kind of nutrient contents, and the mechanism of its processing. Furthermore, numerous factors affecting both the growth of different types of molds and their synthesis for mycotoxins involve humidity, temperature, environment, water activity (a_w_), pH, nutrients, fungal load, physiological state, nature of the substrate, and microbial interaction. Molds commonly gained access to the preserved meats such as basterma and sausage that become active with the prolonged ripening time of such types of processed meat with subsequent mycotoxins production [43, 52].

Our findings evident that the level of aflatoxin B_1_ recovered from examined basterma samples exceeded the international regulatory limits for meat products (> 20 μg/kg). The estimation of aflatoxin B_1_ production is essential during processing and storage. The food-additives, especially spices, are considered a common source of contamination with mycotoxins during meat processing [53]. A previous study reported that more than 80% of the isolated *A. flavus* strains are toxigenic, and 47% of these strains produced aflatoxin B_1_ (chemotypes I and III) [44]. In the present study, the prevalence of aflatoxigenic strains is consistent with the findings of several previous studies [45, 47, 54, 55]. In vitro, *A. flavus* produces pronounced levels of aflatoxins. The emergence of toxigenic *A. flavus* strains calls the need for the application of strict hygienic measures during meat processing and storage [44]. In light of our results, the ochratoxin A detected in four isolated *A. ochraceus* (80%), which is considered a high percentage. However, Zohri et al., 2014 reported that *A. ochraceus* did not produce any detectable amounts of mycotoxins from sausage samples [50]. In the current study, the detected level of ochratoxin A in sausage samples is relatively high and exceeded the legal limits suggested by Italy. Until now, both European and American regulations didn’t set an international legal limit of ochratoxin A in meat and meat products. Only Italy set the legal limit of ochratoxin A as 1 μg/kg meat [56, 57].

In the present study, the macroscopic and microscopic examinations and PCR amplification of the ITS region were used to confirm the diagnosis of the retrieved *A. flavus* and *A. ochraceus* isolates. Furthermore, PCR was performed successfully for the amplification of *aflR1* and *pks* genes. The *aflR1* gene was detected in six *A. flavus* strains isolated from basterma with subsequent sequencing. According to the blastn tool at the NCBI, the identity was 99.73 with query covered 100% percentage to the deposited *aflR1*gene in the Genebank database (accession number MF094441). Besides, The *pks* gene was detected in four *A. ochraceus* strains retrieved from sausage samples and sequenced. According to the blastn tool, the similarity was 100 with a query covered 100% percentage to other *pks* genes deposited in the Genebank database (accession number MF694264). Sequences diversity and omissions in different genes/regions of the aflatoxins and ochratoxins biosynthetic-clusters could be used to detect the polyphyletic grouping of *A. flavus* and *A. ochraceus* [58]. Aflatoxin B is a common carcinogenic mycotoxin commonly produced by *A. flavus*. The aflatoxin regulatory gene (*afl*R) is mainly involved in the regulation of aflatoxins-biosynthesis. The *afl*R gene is encoded for the AflR protein that is responsible for the activation of the functional genes controlling the aflatoxin production pathway. The down-regulation of the *afl*R gene inhibits the expression of other genes and adversely affects the production pathway [59].

Ochratoxin A produced by *A. ochraceus* is known as a potent carcinogenic mycotoxin that is commonly incriminated in chronic interstitial nephritis in humans. Ochratoxin A is a polyketide-derived secondary metabolite. Therefore the polyketide synthase gene (*pks*) is mainly involved in the biosynthesis of ochratoxin A. The molecular detection of the *pks* gene plays a vital role in the demonstration of the ochratoxigenic strains of *A. ochraceus*. The mutant strains of *A. ochraceus* in which the *pks* gene is disturbed or down-regulated were found to miss their ability to produce the ochratoxin A [60].

In conclusion, to the best of our knowledge, this is the first report regarding the HPLC-Molecular-based approaches for the detection of aflatoxin B_1_ and ochratoxin A in processed beef meat in Egypt. The present study emphasized the contamination of processed beef meat products by the toxigenic *A. flavus* and *A. ochraceus* strains that lead to their spoilage. The production of aflatoxin B_1_ and ochratoxin A in processed meat constitutes a public health threat. Aflatoxin B_1_ is commonly associated with basterma samples; furthermore, the ochratoxin A is detected frequently in sausage samples. The routine inspection of mycotoxins in processed meat products is essential to protect human consumers. HPLC is a reliable quantitative assay for the investigation of mycotoxins residues in processed meat products. The combination of both phenotypic and molecular characterization is a reliable epidemiological tool for the identification of the toxigenic *A. flavus* and *A. ochraceus* in meat products. It is necessary to adopt proper hygienic measures during the production and storage of processed meat.

## Data Availability

The datasets used and/or analysed during the current study are available from the corresponding author on reasonable request.
